# Electroacupuncture Decreases Excessive Alcohol Consumption Involving Reduction of FosB/ΔFosB Levels in Reward-Related Brain Regions

**DOI:** 10.1371/journal.pone.0040347

**Published:** 2012-07-09

**Authors:** Jing Li, Yanan Sun, Jiang-Hong Ye

**Affiliations:** 1 Department of Anesthesiology, Pharmacology and Physiology, University of Medicine and Dentistry of New Jersey, New Jersey Medical School, Newark, New Jersey, United States of America; 2 Department of Neurology, Dong-Zhi-Men Hospital, Beijing University of Chinese Medicine, Key Laboratory for Internal Chinese Medicine of Ministry of Education, Beijing, China; Florida State University, United States of America

## Abstract

New therapies are needed for alcohol abuse, a major public health problem in the U.S. and worldwide. There are only three FDA-approved drugs for treatment of alcohol abuse (naltrexone, acamprosate and disulfuram). On average these drugs yield only moderate success in reducing long-term alcohol consumption. Electroacupuncture has been shown to alleviate various drugs of abuse, including alcohol. Although previous studies have shown that electroacupuncture reduced alcohol consumption, the underlying mechanisms have not been fully elucidated. ΔFosB and FosB are members of the Fos family of transcription factors implicated in neural plasticity in drug addiction; a connection between electroacupuncture's treatment of alcohol abuse and the Fos family has not been established. In this study, we trained rats to drink large quantities of ethanol in a modified intermittent access two-bottle choice drinking procedure. When rats achieved a stable baseline of ethanol consumption, electroacupuncture (100 Hz or 2 Hz, 30 min each day) was administered at Zusanli (ST36) for 6 consecutive days. The level of FosB/ΔFosB in reward-related brain regions was assessed by immunohistochemistry. We found that the intake of and preference for ethanol in rats under 100 Hz, but not 2 Hz electroacupuncture regiment were sharply reduced. The reduction was maintained for at least 72 hours after the termination of electroacupuncture treatment. Conversely, 100 Hz electroacupuncture did not alter the intake of and preference for the natural rewarding agent sucrose. Additionally, FosB/ΔFosB levels in the prefrontal cortex, striatal region and the posterior region of ventral tegmental area were increased following excessive ethanol consumption, but were reduced after six-day 100 Hz electroacupuncture. Thus, this study demonstrates that six-day 100 Hz electroacupuncture treatment effectively reduces ethanol consumption and preference in rats that chronically drink excessive amount of ethanol. This effect of electroacupuncture may be mediated by down-regulation of FosB/ΔFosB in reward-related brain regions.

## Introduction

Alcohol abuse is a major public health problem in the U.S. and worldwide. To date, there are only three FDA-approved drugs for treatment of alcohol abuse (naltrexone, acamprosate and disulfuram). On average these drugs yield only moderate success in reducing long-term alcohol consumption [1], [2], [3]. Therefore, new therapies are needed. Acupuncture, consisting of stimulating certain points on the body by means of needles, has been used in China for thousand years. Although it is still not completely clear how the acupuncture signals from the acupoint, such as Zusanli (ST36) are transmitted to the central nervous system, it has been characterized that the afferent impulses induced by acupuncture are mainly transmitted by Aβ and AΔ fibers [4]. Acupuncture activates small myelinated nerve fibers in the muscle, which send impulses to the spinal cord, and then activates three centers (spinal cord, midbrain and pituitary-hypothalamus) and the release of three endorphins (enkephalin, beta endorphin and dynorphin) and other monoamines, eliciting profound physiological effects and self-healing mechanisms [5]. Thus, acupuncture has been regarded widely as an effective mean for some medical conditions, including nausea, pain [6] and drug abuse [7]. Compared with the currently available pharmacological interventions, a clear advantage of acupuncture therapy is that it has the potential to help drug abusers stay away from drugs without major adverse side effects. Previous clinical and preclinical studies have shown that acupuncture or acupuncture combined with electrical stimulation (electroacupuncture, EA) is an effective treatment for alcohol withdrawal syndrome and alcohol abuse [8], [9], . Recently, we have shown that EA of 2 Hz reduced voluntary alcohol intake in rats [14]. However, many questions regarding the basic mechanisms of acupuncture in general and of alcohol abuse in particular have not been well addressed.

Investigations of long-term changes in brain structure and function that accompany chronic exposure to drugs of abuse suggest that alterations in gene regulation contribute substantially to the addictive phenotype [15]. In particular, two transcription factors – ΔFosB and CREB (cAMP responsive element binding protein) have been implicated in addiction-related neural plasticity [15]. The transcription factor Δ FosB, an unusually stable, C-terminally truncated variant of the immediate early gene product FosB, accumulates in the addiction circuitry after most drugs of abuse, including cocaine, morphine, Δ^9^-tetrahydrocannabinol and ethanol [16]. Once expressed, it is relatively stable and can persist in the brain for weeks after the last drug exposure. By regulating numerous genes that are related to dendritic spine architecture and synaptic function and plas­ticity, such as cyclin-dependent kinase 5 and dynorphin [17], [18], ΔFosB mediates the synaptic plasticity which contributes to various behavioural phenotypes in response to drug exposure. Previous studies from our and the other laboratories have shown that chronic alcohol exposures induce the accumulation of ΔFosB within the subregions of the striatum and the prefrontal cortex (PFC) [16], [19], which involves activation of endogenous opioid systems [19]. Given that existing evidence indicates that EA alleviated alcohol consumption and morphine dependence via interacting with opioid receptors [12], [20], [21]; and that acupuncture attenuates stress-induced cocaine relapse by suppressing Fos expression and CREB activation within the subregions of the striatum [22], we hypothesized that EA suppression on alcohol consumption may be mediated by transcription factors, such as FosB/ΔFosB protein in reward-related brain regions. To test this possibility, multiple sessions of EA were administrated at the bilateral acupoint ST36 of rats that chronically drink large quantities of ethanol under a modified intermittent access two-bottle choice drinking procedure (IE). The expression of FosB/ΔFosB in several reward-related brain regions was assessed using immunohistochemistry, which has higher sensitivity than Western blotting and provides greater anatomical details [23].

## Methods

All experiments were performed in accordance with the guidelines of the National Institutes of Health for the Care and Use of Laboratory Animals, and were approved by the Institutional Animal Care and Use Committee of the University of Medicine and Dentistry of New Jersey, Newark, New Jersey.

### Animals and housing

Adult Sprague-Dawley (S-D) rats (250–350 g, at the start of the experiments, Taconic Farm, NY) were individually housed in ventilated cages, in a climate-controlled room (20–22°C), kept on a 12-h light/dark cycle (lights off at 6 p.m.). Food and water were available ad libitum.

### Alcohol drinking procedure

**Figure 1:**
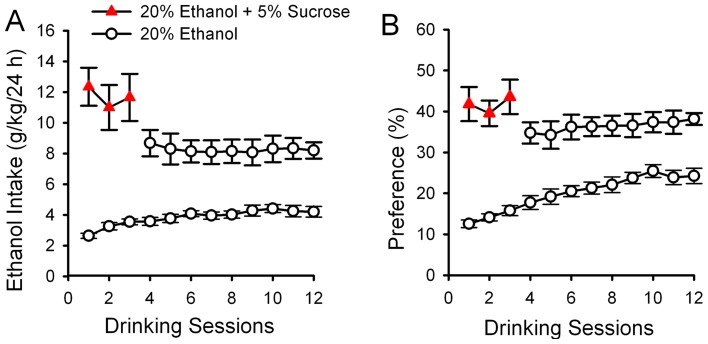
Sucrose induction induced excessive ethanol consumption and high preference in male Sprague-Dawley (SD) rats. The mean (± S.E.M) of ethanol intake (g/kg/24 h) (**A**) and of ethanol preference (**B**) in rats under the intermittent access drinking procedure with 5% sucrose in the first three drinking sessions were much higher than those in rats under the same drinking procedure without sucrose induction (all *p*<0.001). Two-way RM ANOVA followed by Tukey *post hoc* analysis, n = 11 animals for 20% +5% sucrose; n = 22 animals for 20% ethanol.

The animals were first acclimatized to the homecage environment for one week, and were trained to voluntarily drink ethanol under the intermittent access two-bottle choice drinking procedure as described previously [14], [19], [24], [25], [26]. Briefly, animals were given 24-h concurrent access to one bottle of 20% (v/v) ethanol in water and one bottle of water, starting at 6:00 p.m. on Monday. After 24 h, the ethanol bottle was replaced with a second water bottle that was available for the next 24 h. This pattern was repeated on Wednesdays and Fridays. The other days of the week the rats had unlimited access to two bottles of water. The days when ethanol was assessable to the rats, the placement of the ethanol bottle was alternated to control for side preferences. In this study, we modified the procedure by adding 5% sucrose to the 20% ethanol solution in the first three ethanol sessions. This modification rapidly and sharply accelerated ethanol intake (Fig. 1). The amount of ethanol or water consumed was determined by weighing the bottles before access and after 24 h of access. The weight of each rat was measured daily Monday through Friday to monitor health and calculate the grams of ethanol intake per kilogram of body weight. Ethanol consumption was determined by calculating grams of alcohol consumed per kilogram of body weight. The preference ratio of ethanol intake was calculated by the following formula: Preference ratio (%)  =  Ethanol solution intake (ml/24 h)/total fluid intake (ml/24 h ethanol solution + ml/24 h water). Rats were maintained on 20% ethanol intermittent access two-bottle choice procedure for 4 weeks (12 ethanol-access sessions). A bottle containing water in a cage without rats was used to evaluate the spillage due to the experimental manipulations during the test sessions. The spillage was always <1.0 ml (<2.5% of the total fluid intake).

### Blood Ethanol Concentrations (BEC)

On the 13^th^ ethanol drinking session, blood samples were collected from the lateral tail vein of rats (n = 11) following 30 min access to 20% ethanol and water. The samples were centrifuged at 4°C for 15 min at 8000 rpm, and 10 µl plasma from each blood sample was analyzed using nicotinamide adenine dinucleotide-alcohol dehydrogenase (NAD-ADH) enzyme spectrophotometric method [27].

### Sucrose self-administration

To determine whether EA-induced reduction in drinking was selective to alcohol, a separate group (n = 8) of rats were trained to drink 5% sucrose solution under intermittent-access two-bottle choice drinking procedure, similar to that for alcohol drinking. 5% sucrose was selected according to our previous studies [25], [26] and a recent rodent study on the effect of the opioid receptor antagonists, SoRI-9409 on alcohol intake [28]. When SD rats had reached a consistent baseline level after 12 drinking sessions of access to sucrose and water, 100 Hz EA or sham procedures were performed as described below.

### EA treatments

To test the effect of EA on ethanol intake in rats that chronically consumed high amounts of ethanol, 100 Hz EA was administered at bilateral ST36, located near the knee joint of the hind limb, 2 mm lateral to the anterior tubercle of the tibia; EA was applied 30 min each day, 30 min prior to the access to ethanol, for 6 consecutive days (Wednesday through Monday, three consecutive drinking sessions).

All rats were prehandled for 2 min/day for 3 consecutive days prior to EA treatments to reduce stress and facilitate handling. On the test day, under light anesthesia with isoflurane, the rats of both EA and sham groups were lightly restrained, which involved being fixed on a rack with a towel covering their eyes, as described in our recent report [14]. Under these conditions, rats were calm and their limbs and tails could be freely extruded. Two stainless-steel needles with diameter of 0.35 mm and length of 13 mm were inserted about 2–3 mm into ST36 of both legs (for EA group). After the animals woke up from anesthesia, 10 min EA (or sham) was administered. A constant current with square-wave stimulation produced by a programmed pulse generator (Han Actens WQ 1002F, Aeron Optoelectronic Technology Corp., Beijing, China) was given via the two needles for the EA group. The EA frequency was 100 Hz, and the intensity was adjusted to provoke light trembling of muscles (about 0.2–0.3 mA). For the sham group, needles were placed into non-acupoints of the tail, (1/5 tail length from the proximal region of the tail [13], [29]) and no current stimulation was applied. Ethanol (or sucrose) and water intakes were then recorded at 24 h after the onset of drinking. During the 6 treatment days, rats had three ethanol-drinking sessions (day 1, Wednesday; day 3, Friday; and day 6, Monday). Ethanol intake during the drinking session immediately before EA administration and after the last EA administration was recorded respectively as the baseline or post-treatment baseline drinking level. The effects of multiple sessions of low (2 Hz) frequency EA on ethanol intake in rats that chronically consumed high amounts of ethanol was checked in a separate group of rats, in which 2 Hz EA was administered at bilateral ST36 (30 min each day) for six consecutive days. Ethanol and water intakes were recorded as in the above experiment.

### Immunohistochemistry

We have recently reported that chronic ethanol intake induces the accumulation of FosB/ΔFosB in a sub region-specific manner [19]. To determine whether EA-induced reduction in ethanol intake was associated with changes in FosB/ΔFosB expression, we analyzed FosB/ΔFosB immunoreactivity (IR) in reward-related areas of the mesocorticolimbic dopamine system. A group of rats (n = 12) were first trained to drink ethanol using the modified intermittent access two-bottle choice drinking paradigm as described above. When rats voluntarily consumed high amounts of ethanol, they were divided into two subgroups: one (n = 6) received 100 Hz EA at bilateral ST36, the other sham treatment at the tail (n = 6) for 6 consecutive days. During the 6 treatment days, rats had three ethanol-drinking sessions (day 1, Wednesday; day 3, Friday; and day 6, Monday). Rats in the control group (ethanol naïve control, n = 5) were allowed to access to water and food without limitation. There were no significant differences in body weight between the ethanol naïve and ethanol-drinking rats at the end of the experiments.

Ethanol drinking rats treated with EA or sham were sacrificed immediately after the last session of 24 h ethanol access. Ethanol naïve rats were also sacrificed at the same time point. Rats were overdosed with ketamine/xylazine (80 mg/10 mg/kg, i.p.) and transcardially perfused with cold saline followed by 4% paraformaldehyde in 0.1 M sodium phosphate buffer (pH 7.4). Brains were removed, postfixed (2 hours, at 4°C) in the same fixative solution and cryoprotected (overnight at 4°C, 20% sucrose in 0.1 M phosphate buffer, pH 7.4). Serial 30-µm coronal sections of the forebrain were cut on a freezing microtome (Microm HM550, Walldorf, and German), and a 1-in-4 series of brain sections was processed for immunohistochemical detection of FosB/ΔFosB-protein. Sections were incubated in the following series of antibodies: pan-FosB antibody (1∶2000, #sc-48; Santa Cruz Biotechnology, Santa Cruz, California) overnight, at 4°C, biotinylated anti-rabbit IgG (2 hours, 1∶200) (Vector Laboratories, Burlingame, CA). Sections were then incubated in an avidin-biotin-horseradish peroxidase complex solution (45 min) (Vector Elite Kit, Vector Labs, Burlingame, California). Horseradish peroxidase activity was visualized with nickel-diaminobenzidine (Vector Laboratories, Burlingame, CA). Sections from each experimental group were processed simultaneously. Omission of the primary antisera on a subset of sections resulted in a loss of immunoreactivity. Sections were mounted onto chrome-alum slides, dehydrated, and cover slipped.

### Quantization of FosB/ΔFosB immunoreactivity

Changes in FosB/ΔFosB immunoreactivity were measured in sections from prefrontal cortices (PrL, IL and orbitofrontal cortex (OFC)), NAc (core and shell) and dorsolateral striatum (DLS) and dorsomedial striatum (DMS). These brain regions were identified based on the Atlas of Paxinos and Watson [30]. Quantitative measurement was performed using an assisted image analysis system, consisting of an Nikon Eclipse 80i bright field microscope (Micron Optics, Cedar Knoll, NJ) interfaced with a color digital camera Nikon DS-Ri1 (Micron Optics, Cedar Knoll, NJ), and a computer with a NIS-Elements BR 3.0 software (Micron Optics, Cedar Knoll, NJ). Images were obtained at 20×magnification and were averaged from right and left hemispheres in each subject. Two dimensional counts of labeled nuclei from each image [200×images (0.1 mm^2^ area) of FosB/ΔFosB-like immunoreactive nuclei within brain regions of interest] were determined without knowledge of treatment conditions from three separate sections per animal using NIS-Elements BR 3.0 software.

### Statistical Analysis

All data are expressed as mean ± S.E.M. (standard error of the mean). Behavioral data were analyzed with two-way repeated measure ANOVA (RM ANOVA) with the main factors of treatment (EA or sham) and days [baseline (0 day), day 1, 3, 5, postbasline (7 day)]. Tukey *post hoc* analysis was conducted using contrast analysis when day × treatment interaction was *p*<0.05. Immunohistochemical results were analyzed with one-way ANOVA followed by Tukey test.

## Results

### Intermittent access to 20% ethanol (IE) with sucrose added in the first three drinking sessions induces excessive consumption and high preference for ethanol in male SD rats

We previously showed that the IE procedure led the majority of the SD rats to drink moderate levels of ethanol [14]. In this study, we added 5% sucrose in the first three drinking sessions, which greatly increased the amount of ethanol consumed by SD rats that lasted for >3 weeks after sucrose withdrawal (Fig. 1A). Under these conditions, at the 4^th^ to 12^th^ drinking sessions, SD rats consumed 8.2±0.1 g/kg/24 h, which were substantially greater than 4.1±0.1 g/kg/24 h, consumed by rats under the identical procedure but without sucrose induction. Two-way RM ANOVA revealed significant main effects for treatment (*F*_1, 337_ = 269.63, *p*<0.001), day (*F*_11, 337_ = 2.09, *p*<0.05), and treatment × day interaction (*F*_11, 337_ = 6.51, *p*<0.001). *Post-hoc* analysis of the mean amount of ethanol consumed by rats at 4^th^ to 12^th^ drinking sessions showed significant difference between groups with and without sucrose induction.

The preference for ethanol in rats with sucrose induction was also greater than that in those rats without sucrose induction (Fig. 1B). During the 4^th^ to 12^th^ sessions, the mean preference for ethanol was 36.4±0.4% and 22.0±0.8%, respectively for rats with and without sucrose induction. Two-way RM ANOVA revealed significant main effects for treatment (*F*_1, 337_ = 125.73, *p*<0.001) and treatment × time interaction (*F*_11, 337_ = 5.08, *p*<0.001) with strong tendency of time (*F*_11, 337_ = 1.79, *p* = 0.05). *Post-hoc* analysis of the preference for ethanol at 4^th^ to 12^th^ drinking sessions was significantly higher in rats with sucrose than that in rats not given sucrose (all *p*<0.001, Fig. 1B).

We measured BEC of rats from the sucrose induction drinking group described above at the 13th drinking session immediately after the 30-min period of access to ethanol. The BEC ranged from 26.8 to 136.0 mg% with an average of 60.5±10.4 mg%. There was a significant positive correlation between BEC and g/kg ethanol consumed (r^2^ = 0.75, n = 11, *p*<0.001; data not illustrated).

### Multiple sessions of high (100 Hz), but not low frequency (2 Hz) EA treatment reduces excessive consumption of and preference for ethanol, but not sucrose

**Figure 2:**
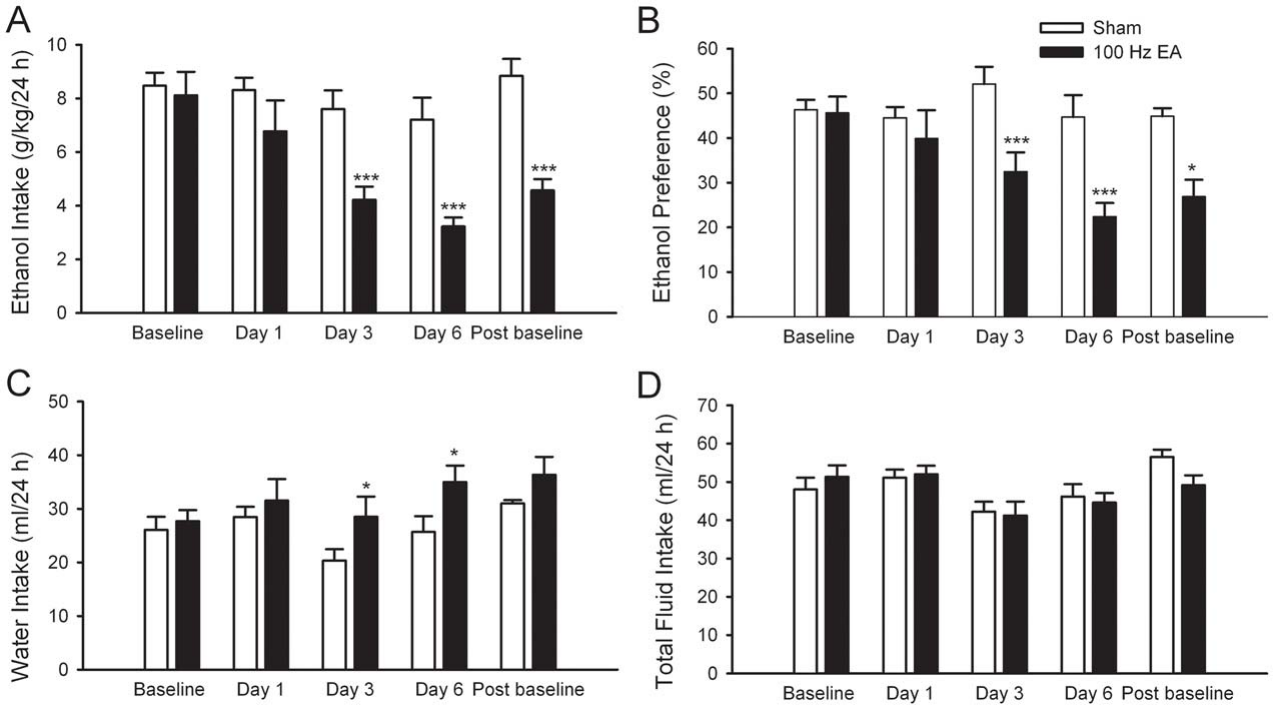
Multiple sessions of high frequency (100 Hz) EA sharply decreased excessive consumption of and preference for ethanol. 100 Hz EA or sham was administered to two different groups of rats for 6 consecutive days, 10 min before the start of ethanol- or water-drinking session. The effect was measured on day 1, 3, and 6. There was no significant difference regarding the baseline drinking levels between the EA and the sham group. Rats in EA group (filled bars) but not in sham group (blank bars) sharply reduced ethanol intake (g/kg/24 h) (**A**) and preference (**B**) for ethanol at the 24 h after the onset of drinking. EA treatment also increased water intake (**C**). Total fluid intake was not affected by multiple EA treatment at 24-h time point on all test days (**D**). The values are expressed as mean ± SEM. *p<0.05, ***p<0.001 compared with sham (two-way RM ANOVA followed by Tukey *post hoc* analysis), n = 8 animals in each group.

A recent rat study found that multiple sessions of high frequency (100 Hz) EA was more effective than the single session of high frequency (100 Hz) in alleviating morphine withdrawal syndrome. Furthermore, the after-effect of multiple sessions EA lasted for at least 7 days [31]. We sought to determine whether multiple sessions of 100 Hz EA treatment can alter ethanol consumption in rats that chronically drink excessive amounts of ethanol. We applied multiple sessions of 100 Hz EA at ST36 or sham to rats when they had reached stable baseline levels of ethanol consumption (see Fig. 1). As shown in Fig. 2A, consecutive 6-day 100 Hz EA, 30 min each day, but not the sham treatment significantly reduced ethanol consumption over the 24-h access period. Two-way RM ANOVA revealed significant main effects of treatment (*F*_1, 51_ = 18.59, *p*<0.001), day (*F*_4,51_ = 9.81, *p*<0.001) and treatment × day interaction (*F*_4,51_ = 5.31, *p* = 0.001). *Post hoc* analysis revealed that ethanol intake over the 24-h access on day 3 and 6 was clearly decreased in the EA treatment group compared with that of sham (all *p*<0.001, Fig. 2A). Remarkably, when EA treatments were terminated on day 6, the reduction was maintained at the 48-72 h drinking session after the last EA administration (*p*<0.01, EA *vs.* sham, Fig. 2A). There was no overall main effect of sham treatment on ethanol intake on all test days compared with baseline drinking levels.

Although the preference ratio for ethanol at 24-h time point examined was not significantly different among groups during the EA-free baseline period, this ratio was significantly reduced after multiple administration of 100 Hz EA. Two-way RM ANOVA revealed significant main effects of treatment (*F*_1,51_ = 11.22, *p* = 0.004), day (*F*_4,51_ = 5.49, *p*<0.001, and the interaction term (*F*_1,51_ = 5.66, *p*<0.001). *Post hoc* analysis revealed that the preference for ethanol at 24-h time point on day 3 and 6 in the multiple sessions of 100 Hz EA treated group was lower than that of sham (all *p*<0.001, Fig. 2B). Furthermore, EA-induced reduction in preference ratio was maintained at the 48–72 h drinking session when EA administrations were terminated (*p*<0.05, EA *vs.* sham, Fig. 2B). There was no overall main effect of sham treatment on the preference for ethanol on test days compared with baseline preference for ethanol (Fig. 2B). Interestingly, consecutive 6-day 100 Hz EA treatment also produced significant effects on water intake (main effect of treatment [*F*_1,51_ = 5.21, *p*<0.05] and day [*F*_4,51_ = 2.97, *p*<0.05], with no effect of treatment × time interaction [*F*_4,51_ = 1.23, *p = *0.31]) (Fig. 2C). Water intake at 24-h time point on day 5 and 7 was significantly increased in EA treated rats compared with sham (*p*<0.05). On all test days, total fluid intake was not affected by consecutive 6-day 100 Hz EA compared with sham treatment (Fig. 2D).

**Figure 3:**
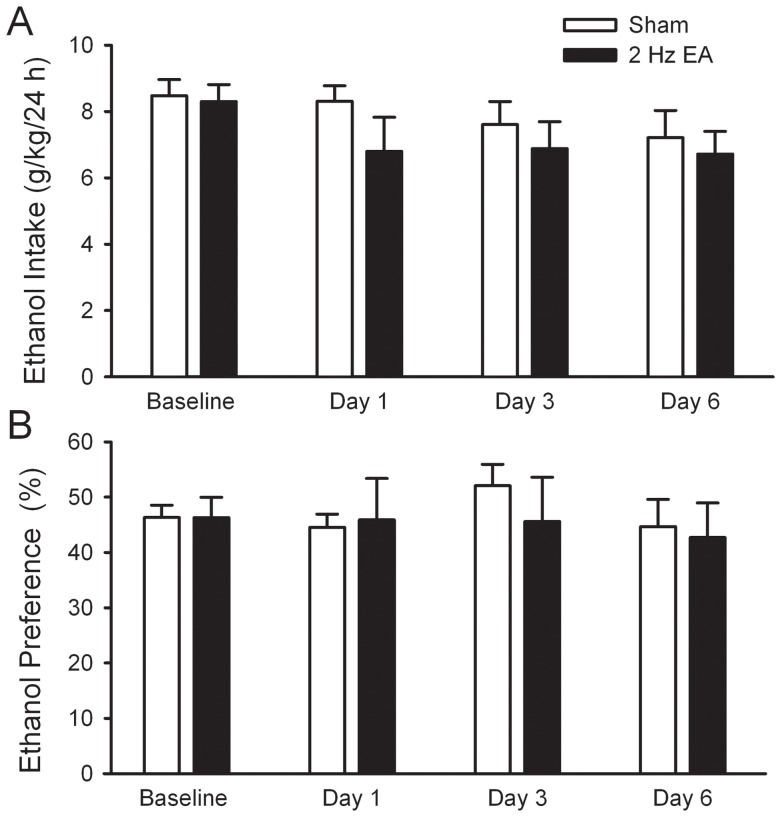
Multiple sessions of low frequency (2 Hz) EA did not alter the excessive intake of and preference for ethanol. EA at low frequency (2 Hz) or sham were administered to rats for 6 consecutive days 10 min before the start of ethanol- or water-drinking session. The effect was measured on day 1, 3, and 6. The values are expressed as mean ± SEM. n = 8 animals in each group.

We previously showed that single low but not high frequency EA reduced moderate ethanol consumption [14]. To determine whether the effect of multiple sessions of EA also depends on its frequency, multiple sessions of low frequency (2 Hz) EA at ST36 was administered to rats that chronically consumed large quantities of ethanol under the IE procedure with sucrose induction as described above. As illustrated in Fig. 3A, under these experimental conditions, multiple sessions of 2 Hz EA did not alter ethanol intake over the 24-h access period on all test days (Fig. 3A). Two-way RM ANOVA for ethanol consumption failed to reveal main effects of treatment (*F*_1, 37_ = 1.43, *p*>0.05), day (*F*_3,37_ = 1.15, *p*>0.05) and treatment × time interaction (*F*_3,37_ = 0.25, *p*>0.05). Accordingly, multiple sessions of 2 Hz EA did not change the preference ratio for ethanol at the 24-h time point on all test days [no main effects of treatment (*F*_1, 37_ = 0.003, *p* = 0.95), day (*F*_3,37_ = 0.54, *p* = 0.65) or treatment × time interaction (*F*_3,37_ = 0.30, *p* = 0.82), Fig. 3B]; or water intake and total fluid (data not shown).

**Figure 4:**
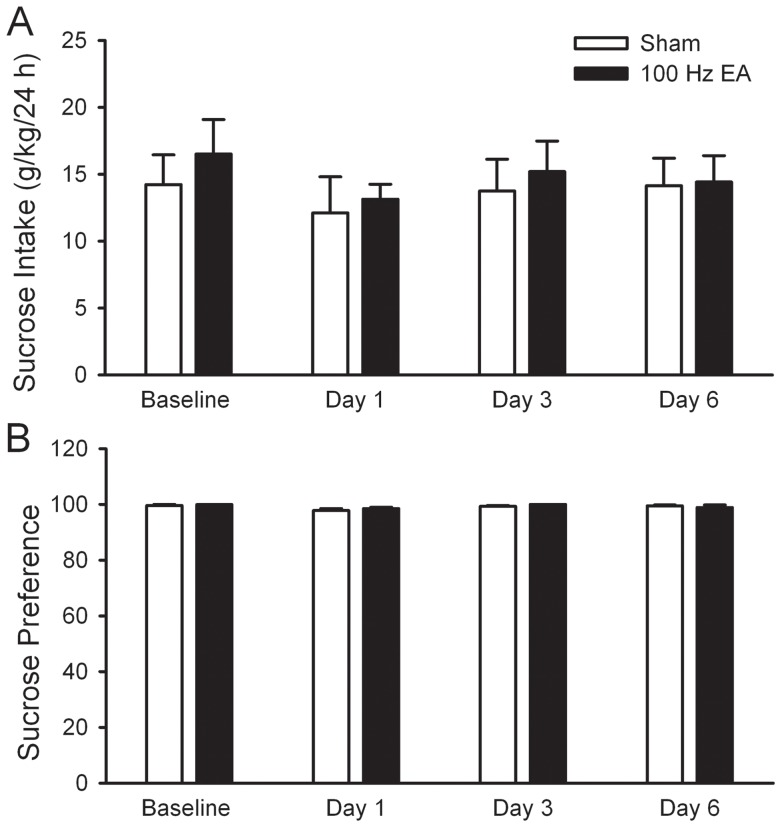
Multiple sessions of 100 Hz EA did not alter the intake of and preference for sucrose. Rats were trained to drink 5% sucrose solution under the intermittent access drinking procedure, similar to that for ethanol drinking. When rats had achieved a consistent baseline level, 100 Hz EA or sham were administered to rats for 6 consecutive days, 10 min before the start of sucrose- or water-drinking session. The effect was measured on day 1, 3, and 6. The values are expressed as mean ± S.E.M. n = 4 animals in each group.

To determine whether the reduction in ethanol consumption induced by multiple sessions of 100 Hz EA is specific to ethanol, we measured the intake of preferred substance sucrose using an intermittent access to 5% sucrose in a two-bottle choice drinking procedure. As shown in Fig. 4, neither sucrose intake nor preference for sucrose at 24-h time point was altered by multiple sessions of 100 Hz EA at ST36. Two-way RM ANOVA for sucrose consumption failed to reveal main effects of treatment (*F*_1, 18_ = 0.23, *p* = 0.65), day (*F*_3,18_ = 1.39, *p* = 0.27) or treatment × time interaction (*F*_3,18_ = 0.19, *p* = 0.90). Furthermore, no significant difference was found between EA and sham for either water consumption or total fluid (data not shown).

### Multiple sessions of 100 Hz EA decreases excessive ethanol consumption-induced accumulation of FosB/ΔFosB in specific reward-related brain regions

**Figure 5:**
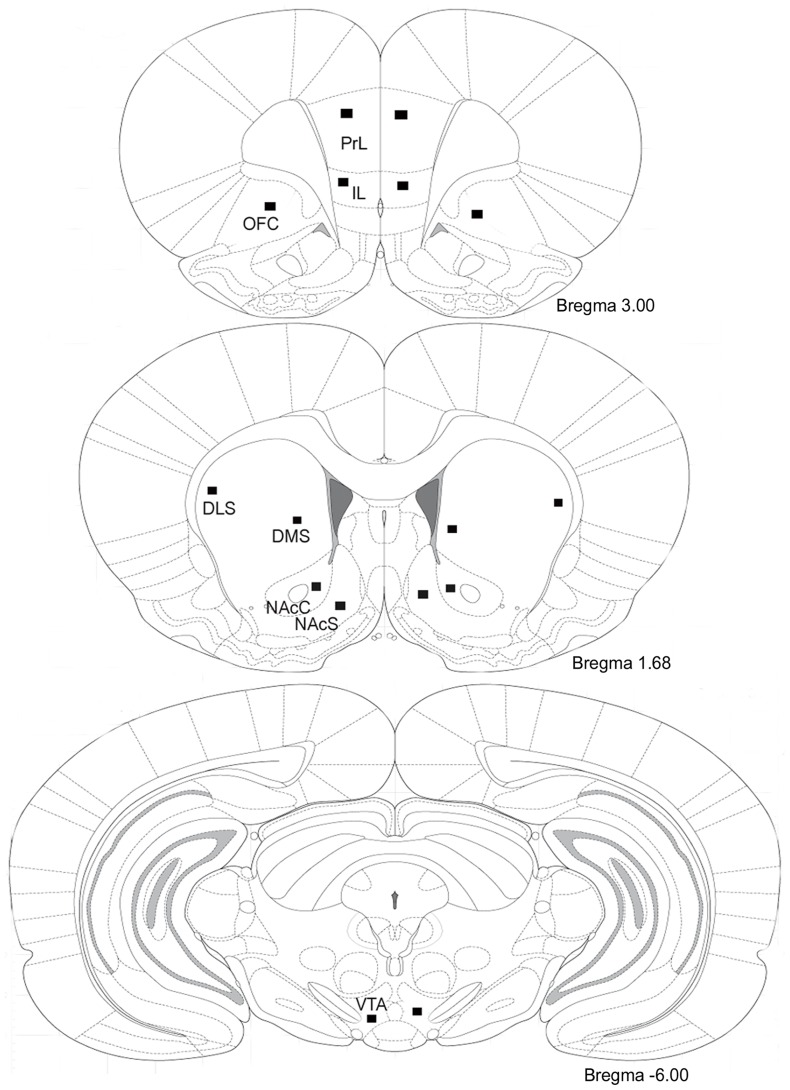
Schematic diagrams of coronal sections of rat brain. The locations that FosB/ΔFosB immunoreactivity was quantified in the following regions: prelimbic cortex (PrL), infralimbic cortex (IL), orbitofrontal cortex (OFC), nucleus accumbens core (NAcC), nucleus accumbens shell (NAcS), dorsolateral striatum (DLS), dorsomedial striatum (DMS), and the ventral tegmental area (VTA). The black squares correspond to a fixed area 200 µm×200 µm in size.

The result**s** described above showed that consecutive 6-day 100 Hz EA selectively lowered the intake of and preference for ethanol in rats that chronically drink excessive amounts of ethanol. Many studies have proposed that the persistent activation of ΔFosB may be a common pathway for addictive disorders [32]. We previously reported that chronic ethanol consumption induces ΔFosB accumulation selectively in the prefrontal cortex and striatal region [19]; dysfunction of these brain reward-related brain regions is associated with ethanol craving and impairment of new learning processes in abstinent alcoholics [33]. Therefore, we assessed FosB/ΔFosB IR in the following reward-related brain regions of the mesocorticolimbic dopamine system (Fig. 5).

#### Striatal region

**Figure 6:**
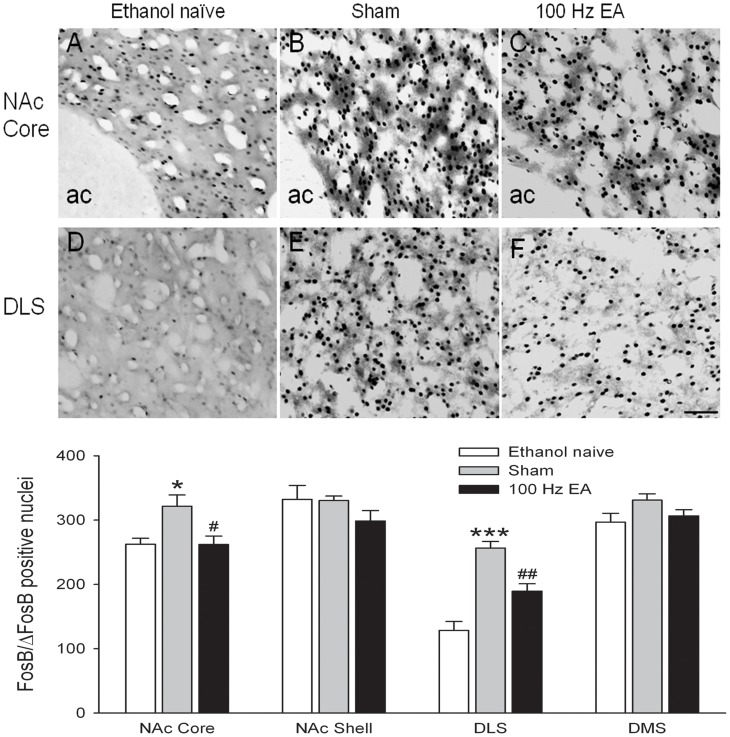
Photomicrographs depicting typical regions of analysis for the NAc core, shell, dorsolateral striatum (DLS) and dorsomedial striatum (DMS). Panels indicate FosB/ΔFosB-positive cells in animals that were drinking water (ethanol naïve, **A, D**), drinking large amounts of ethanol treated with sham (**B, E**), and drinking large amounts of ethanol treated with multiple sessions of 100 Hz EA (**C, F**). Increased numbers of FosB/ΔFosB-positive cells were observed in the NAc core and DLS, but not in the NAc shell and DMS in rats that chronically consumed large amounts of ethanol with sham treatment, as compared with ethanol naive animals. Multiple sessions of 100 Hz EA decreased the accumulation of FosB/ΔFosB in the NAc core and DLS induced by excessive ethanol consumption. Data are expressed as mean ± S.E.M. * *p*<0.05, *** *p*<0.001 indicates a significant difference from ethanol naïve; # *p*<0.05, ## *p*<0.01 indicates a significant difference from sham. Scale bar = 200 µm. ac: anterior commissure.

FosB/ΔFosB expression was regulated differentially within the striatal region as a function of both ethanol and EA treatment. In keeping with our recent report [19], FosB/ΔFosB IR increased robustly within the core of the nucleus accumbens (NAc-Core) and dorsolateral striatum (DLS) (Fig. 6), but not within the dorsal shell (NAc-Shell) and dorsomedial striatum (DMS) in animals that chronically consumed large amounts of ethanol with sham treatment, compared with ethanol naïve controls. Multiple sessions of 100 Hz EA significantly decreased the FosB/ΔFosB IR within the DLS and NAc-Core induced by long-term excessive ethanol consumption (Fig. 6). These observations are supported by one-way ANOVA which revealed a significant main effect of treatment in NAc-Core (*F*_2, 33_ = 6.27, *p* = 0.005) and DLS (*F*_2, 33_ = 28.54, *p*<0.001), but not in NAc-shell (*F*_2, 33_ = 1.36, *p*>0.05) and DMS (*F*_2,33_ = 2.47, *p*>0.05).

#### Prefrontal cortex

**Figure 7:**
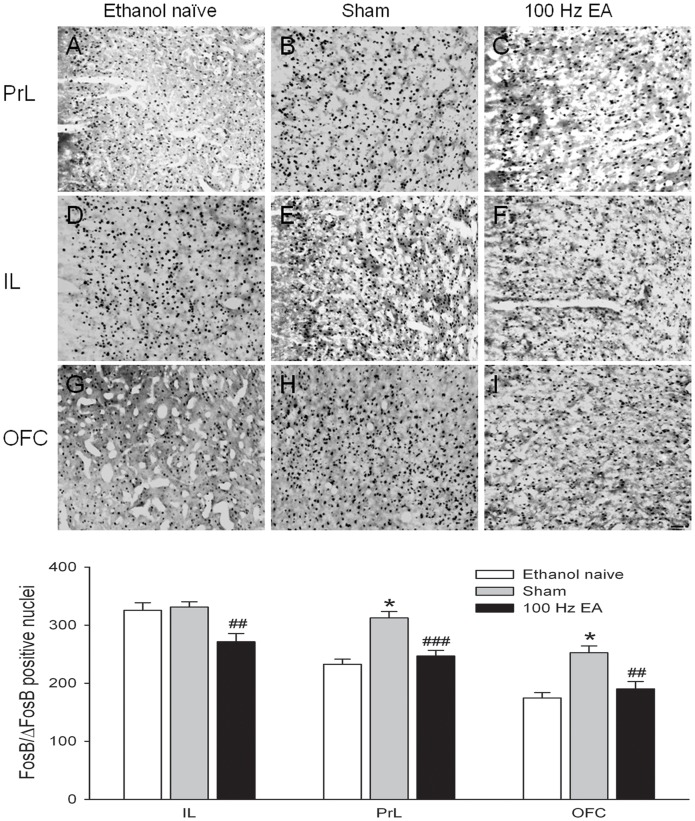
Photomicrographs depicting typical brain regions of analysis for the prelimbic (PrL), infralimbic (IL) and the orbitofrontal cortex (OFC) divisions of the prefrontal cortex. Panels indicate FosB/ΔFosB-positive cells in animals that were drinking water (ethanol naïve, **A, D** and **G**), drinking large amounts of ethanol treated with sham (**B, E** and **H**), and drinking large amounts of ethanol treated with multiple high frequency EA (**C, F** and **I**). Increased numbers of FosB/ΔFosB-positive cells were observed in the PrL and OFC, but not in the IL in rats that chronically consumed large amounts of ethanol with sham treatment, as compared with ethanol naive animals. Multiple sessions of 100 Hz EA significantly decreased the numbers of FosB/ΔFosB positive nuclei in the IL, PrL and OFC in rats that consumed large amounts of ethanol. Data are expressed as mean ± S.E.M. * indicates significant difference from ethanol naïve (*p*<0.001); ## *p*<0.01, ### *p*<0.001 indicates a significant difference from sham. Scale bar = 100 µm.

We recently reported that chronic ethanol exposure robustly increases FosB/ΔFosB IR in orbitofrontal cortex (OFC), but not the medial prefrontal cortex [19]. However, in the current study, FosB/ΔFosB IR was significantly increased in both of the prelimbic area of the prefrontal cortex (PrL) and OFC in animals that chronically consumed large amounts of ethanol with sham treatment (Fig. 7, *p*<0.001 sham *vs.* ethanol naïve). No statistical differences in FosB/ΔFosB-positive nuclei counts were found between the sham group and ethanol naïve control group in the infralimbic area of the prefrontal cortex (IL). Multiple sessions of 100 Hz EA significantly decreased FosB/ΔFosB IR in the PrL, the OFC and the IL (all *p*<0.01 EA group *vs.* sham). Accordingly, one-way ANOVA yielded a significant main effect of treatment in IL (*F*_2, 33_ = 7.06, *p* = 0.003), PrL (*F*_2, 33_ = 18.61, *p*<0.001) and OFC (*F*_2, 33_ = 13.23, *p*<0.001).

#### The ventral tegmental area (VTA)

**Figure 8:**
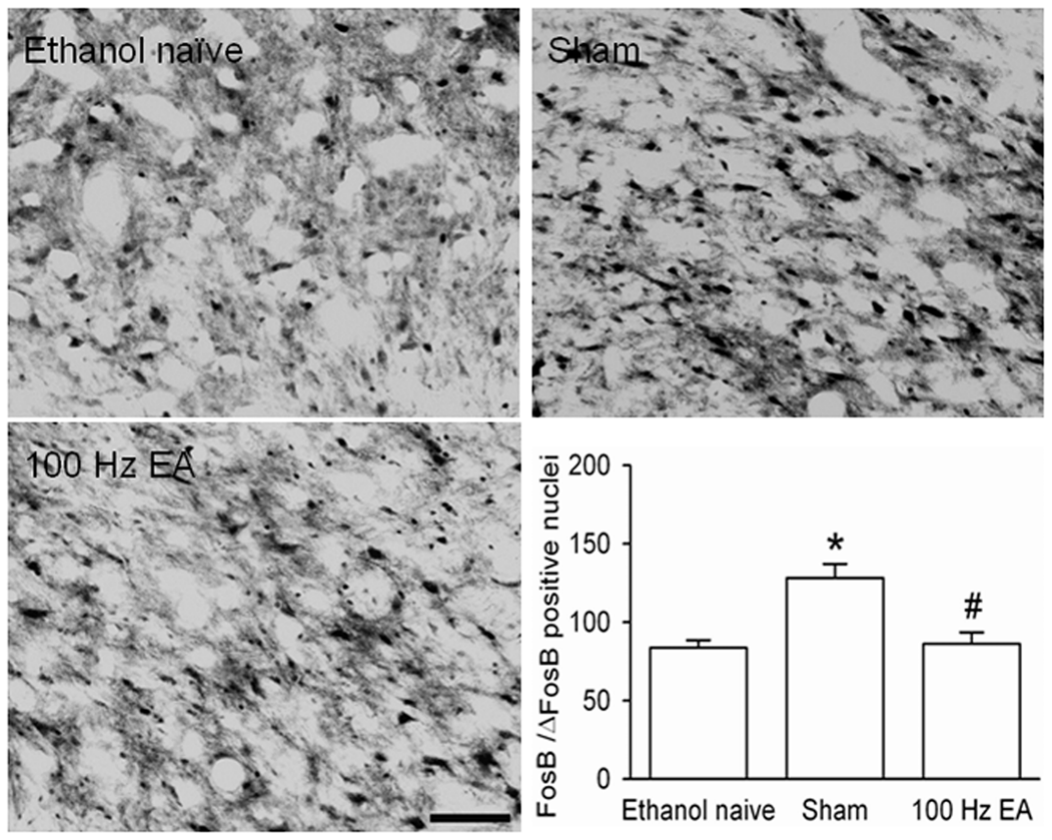
Photomicrographs depicting typical brain regions of analysis for the posterior VTA. Panels indicate FosB/ΔFosB-positive cells in animals that were drinking water (ethanol naïve), drinking large amounts of ethanol treated with sham (Sham), and drinking large amounts of ethanol treated with multiple sessions of 100 Hz EA (100 Hz EA). Increased numbers of FosB/ΔFosB-positive nuclei were observed in the posterior VTA in rats that chronically consumed large amounts of ethanol with sham treatment, as compared with ethanol naive animals. Multiple sessions of 100 Hz EA decreased the accumulation of FosB/ΔFosB in the VTA induced by excessive ethanol consumption. Data are expressed as mean ± S.E.M. * indicates significant difference from ethanol naïve (*p*<0.001), #, a significant difference from sham (*p*<0.001). Scale bar = 200 µm.

VTA is believed to be a critical brain region of reward. A recent study showed that chronic psychostimulant administration induces the accumulation of ΔFosB, specifically in the posterior tail of the VTA [23], [34]. As illustrated in Fig. 8, FosB/ΔFosB IR in the posterior region of the VTA (Bregma − 5.20 mm to Bregma −6.8 mm) was robustly increased in rats that chronically consumed large amounts of ethanol compared to that in ethanol naïve group (*p*<0.001), and was substantially reduced after multiple sessions of EA (*p*<0.001, EA group *vs.* sham) but not after sham treatment. There was an overall main effect of treatment on FosB/ΔFosB IR in the posterior VTA (*F*_2,33_ = 12.04, *p*<0.001, Fig. 8).

## Discussion

We reported here that six-day 100 Hz EA (30 min each day) at the bilateral acupoint ST36 selectively reduced excessive consumption of and preference for ethanol over 24 h access period. The reduction maintained for at least 72 h after the termination of EA treatment. Furthermore, this EA regiment decreased FosB/ΔFosB expression induced by chronic ethanol exposure in the reward-related brain regions.

We have recently reported that a single 20 min low (2 Hz), but not high (100 Hz) frequency EA at ST36 lowered the moderate intake of ethanol in SD rats [14]. There is recent evidence that multiple sessions of high frequency EA are more effective than the single session of high frequency EA in alleviating morphine withdrawal syndrome [31]. In this study we investigated whether 100 Hz EA was effective in reducing excessive ethanol consumption in rats under a modified IE drinking procedure, in which all rats consumed high amounts of ethanol (8.2±0.1 g/kg) with high preference ratio for ethanol (36.4±0.4%). Although we did not monitor withdrawal signs in the current study, we have previously reported that rats under the similar drinking program did show mild withdrawal signs [14], [25]. The BEC of rats in the current study was 60.5±10.4 mg%, which was almost two times higher than that of SD rats [14] and similar to that of Long-Evans rats [25] which showed withdrawal signs. Thus, it is highly possible that rats under the current experimental conditions may develop ethanol dependence and have signs of withdrawal. Remarkably, consecutive six-day 100 Hz EA reduced free ethanol intake by almost half over the 24-h access period. Intriguingly, this EA regiment also significantly enhanced water intake, suggesting that these animals were going to the direction opposite to ethanol drinking. Importantly, the reduction on ethanol intake was maintained for >72 h after the termination of EA treatments. Conversely, multiple sessions of 100 Hz EA did not change the intake of and preference for the natural reward substance sucrose. Our current finding is in line with a recent study showing that multiple sessions of EA suppressed the morphine withdrawal syndrome, which was maintained for at least 7 days in a treatment-free period. This long-lasting effect of EA may be attributable to the increased dynorphin, since multiple sessions of 100 Hz EA is very efficient in accelerating the biosynthesis of dynorphin, as reflected by the prompt up-regulation of preprodynorphin mRNA, which was maintained for at least 7 days in the brain [31].

Previous evidence indicates that dynorphin released in CNS, via interacting with κ-opioid receptor (KOR) plays an important role in 100 Hz EA-induced suppression of morphine withdrawal syndrome [20], [35], [36]. Furthermore, multiple sessions of 100 Hz EA were shown to be more effective than single EA in blocking the down-regulation of preprodynorphin mRNA induced by chronic morphine exposure [31]. It has been demonstrated that dynorphin-κ opioid systems may have an important role in driving compulsive drug intake [37]. The expression of dynorphin and KORs were lower in rodents who consumed high levels of ethanol than their low drinking counterparts [38], [39], [40]. Furthermore, systemic administration of the KOR agonist U50,488H in rats significantly reduces ethanol intake [41], whereas administration of the KOR antagonist increases ethanol intake [42]. Importantly, polymorphisms in dynorphin and the KOR have been associated with increased risk of alcoholism in humans [43]. Taken together, these data suggest a modulatory role of dynorphin over ethanol drinking, in which the dynorphin/KOR system functions to reduce ethanol drinking. Since 100 Hz EA can specifically facilitate dynorphin release [44], we hypothesized that dynorphin mediates at least in part of the reduction in ethanol consumption induced by 100 Hz EA observed in the current study. However, the mechanism underlying the dynorphin mediated reduction in ethanol consumption is not completely clear. A reduction in ethanol consumption induced by the increase of dynorphin was also observed in previous studies [45], [46]. These authors interpreted their data to indicate that dynorphin may provide postingestive feedback to regulate subsequent ethanol consumption bouts.

Predynorphin gene is one of the transcriptional targets for ΔFosB [18], [32]. Over-expression of ΔFosB in the NAc and the dorsal striatum increased locomotor activity and rewarding responses to morphine partly through the suppression of dynorphin expression [18]. Since 100 Hz EA accelerated the release of dynorphin in the spinal cord, and restored the expression of prodynorphin gene in the brain in morphine dependent animal [31], [36], we propose that 100 Hz EA may alter the function of ΔFosB in the brain.

This study shows that ΔFosB transcription factors in the prefrontal cortex, the striatal region and the posterior VTA may play an important role in 100 Hz EA-induced reduction of excessive ethanol intake. It highlights that EA-induced reduction of ethanol consumption may involve diverse pathways that contribute to ethanol drinking, including cognitive, motivational and motor neural circuits. The NAc core may contribute to conditioned stimulus-supported drug-seeking behavior [47]. Likewise, dorsal striatum may contribute to the compulsive or habit-like nature of drug consumption [48]. The lateral (DLS) and medial (DMS) parts of dorsal striatum have distinct anatomical inputs and outputs and therefore different functions [49]. For instance, endogenous brain-derived neurotrophic factor in the DLS but not in the DMS controls voluntary ethanol intake [50]. In line with these findings, we showed that chronic ethanol self-administration induced pronounced accumulation of ΔFosB in the NAc core and the DLS, but not in the NAc shell and the DMS. Importantly, we further showed that multiple sessions of 100 Hz EA significantly attenuated the accumulation of FosB/ΔFosB IR in the NAc core and the DLS induced by excessive ethanol consumption, and the attenuation was more significant in DLS than in NAc core.

The PFC is responsible for executive function, decision-making, and the implementation of goal-directed actions. Subregions of the PFC include the PrL that guides response initiation and the IL that mediates response inhibition; both regions guide actions and outcomes. The PrL and IL may serve as on-off mechanisms in both conditioned drug and fear responses. Furthermore, the subregion of OFC is a key brain region implicated in regulating goal-directed behavior and impulsivity [51]. Accumulation of ΔFosB in the PFC is thought to be directly involved in addiction maintenance by producing tolerance to the cognitive disrupting effects of drugs via its actions in the PFC [52]. During drug withdrawal, overexpression of ΔFosB enhances impulsivity, which further promotes drug self-administration [53], [54]. Importantly, genetic or viral overexpression of ΔJunD -a dominant negative mutant of JunD that antagonizes ΔFosB and other AP1-mediated transcriptional activity – in the OFC blocks these key effects of drug exposure [53]. The current study showed that excessive ethanol intake induced high levels of ΔFosB in PrL and OFC, which were blocked by multiple sessions of 100 Hz EA; furthermore, although FosB/ΔFosB IR in the IL was not altered by excessive ethanol consumption, it was reduced by multiple sessions of 100 Hz EA.

The VTA, the origin of mesolimbic dopamine system, is critical for motivated behaviors of abused drugs, including ethanol. Previously Perrotti and colleagues have provided evidence that following acute or chronic exposure to several psychostimulant such as cocaine and amphetamine, the expression of FosB/ΔFosB in the posterior/tail VTA was increased. Importantly, the expression is essentially presented in the GABAergic neurons, with no detectable expression in the DA neurons [23], [55]. In keeping with Perrotti's finding, we found that following chronic ethanol exposure, the expression of FosB/ΔFosB in the posterior/tail VTA was increased. Although we did not identify the cell types where FosB/ΔFosB was expressed in the present study, based on Perrotti's finding described above, we speculate that FosB/ΔFosB may be expressed in the GABAergic neurons; although the mechanism of ΔFosB induction solely in the subset of GABA neurons of posterior VTA is still unclear. Given that repeated administration of a dopamine uptake inhibitor induced FosB/ΔFosB in the VTA [23], it has been suggested that dopamine system mediates ΔFosB induction. Interestingly, six-day 100 Hz EA significantly decreased FosB/ΔFosB IR in the posterior/tail VTA. Given that activation of KORs in the VTA can hyperpolarize DA neurons and suppress dopamine release by direct actions at the release site [56], we propose that by the activation of KORs, multiple sessions of 100 Hz EA might suppress dopamine release, which leads to the inhibition of FosB/ΔFosB expression in the VTA GABA neurons [23], although the consequence of GABA neuron inhibition requires further investigation. Furthermore, recent studies have demonstrated that KORs are functionally expressed on the PFC- and amygdala- but not NAc-projecting DA neurons in the VTA [57], [58]; thus, the inhibition of alcohol intake induced by multiple sessions of 100 Hz EA could be due to selective inhibition of the VTA-PFC or VTA-amygdala DA circuits or both via selective activation of KORs in the VTA.

In summary, this study shows that multiple sessions of EA at high frequency (100 Hz) at acupoint ST36 are effective in reducing (1) ethanol consumption and preference in rats that chronically consumed high amounts of ethanol, and (2) FosB/ΔFosB IR in reward-related brain regions. Given that the ΔFosB and FosB are important members of the Fos family of transcription factors implicated in neural plasticity in drug addiction, it appears that ΔFosB and FosB in the reward-related brain regions are key players of EA action in reducing excessive alcohol consumption.

## References

[1] Anton RF, O'Malley SS, Ciraulo DA, Cisler RA, Couper D (2006). Combined pharmacotherapies and behavioral interventions for alcohol dependence: the COMBINE study: a randomized controlled trial.. JAMA.

[2] Mann K, Lehert P, Morgan MY (2004). The efficacy of acamprosate in the maintenance of abstinence in alcohol-dependent individuals: results of a meta-analysis.. Alcohol Clin Exp Res.

[3] Meyers RJ, Smith JE, Lash DN (2003). The Community Reinforcement Approach.. Recent Dev Alcohol.

[4] Lu GW (1983). Characteristics of afferent fiber innervation on acupuncture points zusanli.. Am J Physiol.

[5] Stux G (1987). Acupuncture text book and atlas.. Berlin: Springer-Verlag.

[6] Jindal V, Ge A, Mansky PJ (2008). Safety and efficacy of acupuncture in children: a review of the evidence.. J Pediatr Hematol Oncol.

[7] Han J, Cui C, Wu L (2011). Acupuncture-related techniques for the treatment of opiate addiction: a case of translational medicine.. Front Med.

[8] Yoshimoto K, Kato B, Sakai K, Shibata M, Yano, et al (2001). Electroacupuncture stimulation suppresses the increase in alcohol-drinking behavior in restricted rats.. Alcohol Clin Exp Res.

[9] Karst M, Passie T, Friedrich S, Wiese B, Schneider U (2002). Acupuncture in the treatment of alcohol withdrawal symptoms: a randomized, placebo-controlled inpatient study.. Addict Biol.

[10] Kim YH, Schiff E, Waalen J, Hovell M (2005). Efficacy of acupuncture for treating cocaine addiction: a review paper.. J Addict Dis.

[11] Kunz S, Schulz M, Lewitzky M, Driessen M, Rau H (2007). Ear acupuncture for alcohol withdrawal in comparison with aromatherapy: a randomized-controlled trial.. Alcohol Clin Exp Res.

[12] Overstreet DH, Cui CL, Ma YY, Guo CY, Han JS (2008). Electroacupuncture reduces voluntary alcohol intake in alcohol-preferring rats via an opiate-sensitive mechanism.. Neurochem Res.

[13] Yang CH, Lee BB, Jung HS, Shim I, Roh PU (2002). Effect of electroacupuncture on response to immobilization stress.. Pharmacol Biochem Behav.

[14] Li J, Zou Y, Ye JH (2011). Low frequency electroacupuncture selectively decreases voluntarily ethanol intake in rats.. Brain Res Bull.

[15] Robison AJ, Nestler EJ (2011). Transcriptional and epigenetic mechanisms of addiction.. Nat Rev Neurosci.

[16] Perrotti LI, Weaver RR, Robison B, Renthal W, Maze I (2008). Distinct patterns of DeltaFosB induction in brain by drugs of abuse.. Synapse.

[17] Bibb JA, Chen J, Taylor JR, Svenningsson P, Nishi A (2001). Effects of chronic exposure to cocaine are regulated by the neuronal protein Cdk5.. Nature.

[18] Zachariou V, Bolanos CA, Selley DE, Theobald D, Cassidy MP (2006). An essential role for DeltaFosB in the nucleus accumbens in morphine action.. Nat Neurosci.

[19] Li J, Cheng Y, Bian WL, Liu X, Zhang C (2010). Region-specific induction of FosB/deltaFosB by voluntary alcohol intkae: effects of naltrexone.. Alcohol Clin Exp Res 34.

[20] Cui CL, Wu LZ, Luo F (2008). Acupuncture for the treatment of drug addiction.. Neurochem Res.

[21] Yang CH, Yoon SS, Hansen DM, Wilcox JD, Blumell BR (2010). Acupuncture inhibits GABA neuron activity in the ventral tegmental area and reduces ethanol self-administration.. Alcohol Clin Exp Res.

[22] Yoon SS, Yang EJ, Lee BH, Jang EY, Kim HY (2012). Effects of acupuncture on stress-induced relapse to cocaine-seeking in rats.. Psychopharmacology.

[23] Perrotti LI, Bolanos CA, Choi KH, Russo SJ, Edwards S (2005). DeltaFosB accumulates in a GABAergic cell population in the posterior tail of the ventral tegmental area after psychostimulant treatment.. Eur J Neurosci.

[24] Simms JA, Steensland P, Medina B, Abernathy KE, Chandler LJ (2008). Intermittent access to 20% ethanol induces high ethanol consumption in Long-Evans and Wistar rats.. Alcohol Clin Exp Res.

[25] Li J, Bian W, Dave V, Ye JH (2011). Blockade of GABA(A) receptors in the paraventricular nucleus of the hypothalamus attenuates voluntary ethanol intake and activates the hypothalamic-pituitary-adrenocortical axis.. Addict.

[26] Li J, Nie H, Bian W, Dave V, Janak PH (2012). Microinjection of Glycine into the Ventral Tegmental Area Selectively Decreases Ethanol Consumption.. J Pharmacol Exp.

[27] Poklis A, Mackell MA (1982). Evaluation of a modified alcohol dehydrogenase assay for the determination of ethanol in blood.. Clin Chem.

[28] Nielsen CK, Simms JA, Pierson HB, Li R, Saini SK (2008). A novel delta opioid receptor antagonist, SoRI-9409, produces a selective and long-lasting decrease in ethanol consumption in heavy-drinking rats.. Biol Psychiatry.

[29] Zhao RJ, Yoon SS, Lee BH, Kwon YK, Kim KJ (2006). Acupuncture normalizes the release of accumbal dopamine during the withdrawal period and after the ethanol challenge in chronic ethanol-treated rats.. Neurosci Lett.

[30] Paxinos G, Watson C (2007). The Rat brain in stereotaxic coordinates 6th edition, Academic press..

[31] Wang GB, Wu LZ, Yu P, Li YJ, Ping XJ (2011). Multiple 100 Hz electroacupuncture treatments produced cumulative effect on the suppression of morphine withdrawal syndrome: Central preprodynorphin mRNA and p-CREB implicated.. Peptides.

[32] Nestler EJ (2005). Is there a common molecular pathway for addiction?. Nat Neurosci.

[33] Chen G, Cuzon Carlson VC, Wang J, Beck A, Heinz A (2011). Striatal involvement in human alcoholism and alcohol consumption, and withdrawal in animal models.. Alcohol Clin Exp Res.

[34] Kaufling J, Veinante P, Pawlowski SA, Freund-Mercier MJ, Barrot M (2009). Afferents to the GABAergic tail of the ventral tegmental area in the rat.. J Comp Neurol.

[35] Shi XD, Wang GB, Ma YY, Ren W, Luo F (2004). Repeated peripheral electrical stimulations suppress both morphine-induced CPP and reinstatement of extinguished CPP in rats: accelerated expression of PPE and PPD mRNA in NAc implicated.. Brain Res Mol Brain Res.

[36] Wu LZ, Cui CL, Tian JB, Ji D, Han JS (1999). Suppression of morphine withdrawal by electroacupuncture in rats: dynorphin and kappa-opioid receptor implicated.. Brain Res.

[37] Wee S, Koob GF (2010). The role of the dynorphin-kappa opioid system in the reinforcing effects of drugs of abuse.. Psychopharmacology (Berl).

[38] Fadda P, Tronci S, Colombo G, Fratta W (1999). Differences in the opioid system in selected brain regions of alcohol-preferring and alcohol-nonpreferring rats.. Alcohol Clin Exp Res.

[39] Marinelli PW, Kiianmaa K, Gianoulakis C (2000). Opioid propeptide mRNA content and receptor density in the brains of AA and ANA rats.. Life Sci.

[40] Winkler A, Spanagel R (1998). Differences in the kappa opioid receptor mRNA content in distinct brain regions of two inbred mice strains.. Neuroreport.

[41] Lindholm S, Werme M, Brene S, Franck J (2001). The selective kappa-opioid receptor agonist U50,488H attenuates voluntary ethanol intake in the rat.. Behav Brain Res.

[42] Mitchell JM, Liang MT, Fields HL (2005). A single injection of the kappa opioid antagonist norbinaltorphimine increases ethanol consumption in rats.. Psychopharmacology (Berl).

[43] Xuei X, Dick D, Flury-Wetherill L, Tian HJ, Agrawal A (2006). Association of the kappa-opioid system with alcohol dependence.. Mol Psychiatry.

[44] Han JS (2003). Acupuncture: neuropeptide release produced by electrical stimulation of different frequencies.. Trends Neurosci.

[45] Logrip ML, Janak PH, Ron D (2008). Dynorphin is a downstream effector of striatal BDNF regulation of ethanol intake.. FASEB J.

[46] Logrip ML, Janak PH, Ron D (2009). Blockade of ethanol reward by the kappa opioid receptor agonist U50,488H.. Alcohol.

[47] Everitt BJ, Robbins TW (2005). Neural systems of reinforcement for drug addiction: from actions to habits to compulsion.. Nat Neurosci.

[48] Vanderschuren LJ, Di Ciano P, Everitt BJ (2005). Involvement of the dorsal striatum in cue-controlled cocaine seeking.. J Neurosci.

[49] Voorn P, Vanderschuren LJ, Groenewegen HJ, Robbins TW, Pennartz CM (2004). Putting a spin on the dorsal-ventral divide of the striatum.. Trends Neurosci.

[50] Jeanblanc J, He DY, Carnicella S, Kharazia V, Janak PH (2009). Endogenous BDNF in the dorsolateral striatum gates alcohol drinking.. J Neurosci.

[51] Krawczyk DC (2002). Contributions of the prefrontal cortex to the neural basis of human decision making.. Neurosci Biobehav Rev.

[52] Nestler EJ (2008). Review. Transcriptional mechanisms of addiction: role of DeltaFosB.. Philos Trans R Soc Lond B Biol Sci.

[53] Winstanley CA, LaPlant Q, Theobald DE, Green TA, Bachtell RK (2007). DeltaFosB induction in orbitofrontal cortex mediates tolerance to cocaine-induced cognitive dysfunction.. J Neurosci.

[54] Winstanley CA, Green TA, Theobald DE, Renthal W, LaPlant Q (2009). DeltaFosB induction in orbitofrontal cortex potentiates locomotor sensitization despite attenuating the cognitive dysfunction caused by cocaine.. Pharmacol Biochem Behav.

[55] Kaufling J, Waltisperger E, Bourdy R, Valera A, Veinante P (2010). Pharmacological recruitment of the GABAergic tail of the ventral tegmental area by acute drug exposure.. Br J Pharmacol.

[56] Ford CP, Beckstead MJ, Williams JT (2007). Kappa opioid inhibition of somatodendritic dopamine inhibitory postsynaptic currents.. J Neurophysiol.

[57] Margolis EB, Lock H, Chefer VI, Shippenberg TS, Hjelmstad GO (2006). Kappa opioids selectively control dopaminergic neurons projecting to the prefrontal cortex.. Proc Natl Acad Sci U S A.

[58] Margolis EB, Mitchell JM, Ishikawa J, Hjelmstad GO, Fields HL (2008). Midbrain dopamine neurons: projection target determines action potential duration and dopamine D(2) receptor inhibition.. J Neurosci.

